# Thermo-Plasmonic Killing of *Escherichia coli* TG1 Bacteria

**DOI:** 10.3390/ma12091530

**Published:** 2019-05-10

**Authors:** Ferdinanda Annesi, Alfredo Pane, Maria Adele Losso, Alexa Guglielmelli, Fabrizio Lucente, Francesca Petronella, Tiziana Placido, Roberto Comparelli, Maria Grazia Guzzo, Maria Lucia Curri, Roberto Bartolino, Luciano De Sio

**Affiliations:** 1 CNR-Lab. Licryl, Institute NANOTEC, 87036 Arcavacata di Rende, Italy; ferdinanda.annesi@cnr.it (F.A.); alfredo.pane@cnr.it (A.P.); alexa.guglielmelli@unical.it (A.G.); roberto.bartolino@fis.unical.it (R.B.); 2 Department DiBEST (Biology, Ecology and Earth Sciences), University of Calabria, 87036 Arcavacata di Rende, Italy; maria_adele.losso@unical.it (M.A.L.); fabrizio.lucente93@gmail.com (F.L.); mariagraziaguzzo22@gmail.com (M.G.G.); 3 Department of Physics, University of Calabria, Arcavacata di Rende, 87036 Cosenza, Italy; 4 CNR-IPCF, National Research Council of Italy, Institute for Physical and Chemical Processes-Bari Division, Via Orabona 4, I-70126 Bari, Italy; f.petronella@ba.ipcf.cnr.it (F.P.); t.placido@ba.ipcf.cnr.it (T.P.); r.comparelli@ba.ipcf.cnr.it (R.C.); lucia.curri@ba.ipcf.cnr.it (M.L.C.); 5 Department of Chemistry, “A. Moro” University of Bari, Via Orabona 4, I-70126 Bari, Italy; 6 Department of Medico-surgical Sciences and Biotechnologies, Sapienza University of Rome, Corso della Repubblica 79, 04100 Latina, Italy

**Keywords:** nanomaterials, plasmonics, bacteria disinfection, photothermal effects, optics

## Abstract

Plasmonic photo-thermal therapy (PPTT) is a minimally invasive, drug-free, therapy based on the properties of noble metal nanoparticles, able to convert a bio-transparent electromagnetic radiation into heat. PPTT has been used against cancer and other diseases. Herein, we demonstrate an antimicrobial methodology based on the properties of gold nanorods (GNRs). Under a resonant laser irradiation GNRs become highly efficient light to heat nano-converters extremely useful for PPTT applications. The concept here is to assess the antimicrobial effect of easy to synthesize, suitably purified, water-dispersible GNRs on *Escherichia coli* bacteria. A control on the GNRs concentration used for the process has been demonstrated critical in order to rule out cytotoxic effects on the cells, and still to be able to generate, under a near infrared illumination, an adequate amount of heat suited to increase the temperature up to ≈50 °C in about 5 min. Viability experiments evidenced that the proposed system accomplished a killing efficiency suitable to reducing the *Escherichia coli* population of about 2 log CFU (colony-forming unit).

## 1. Introduction

Bacterial adhesion and proliferation represent an extremely critical problem to human and animal health. To date, antibiotics are the best treatment for bacterial infections [1]. They act at different levels by inhibiting DNA replication, translation or synthesis of the cell membrane. On the other hand, resistant strains are the main consequences of their large use. Most pathogen bacteria bind to a surface and form a structured biofilm, where many species interact with each other and get their natural habitat. Biofilms are present in mammals as a result of alterations of different organs such as skin, nose, lung and intestine, as well as on abiotic surfaces, including medical devices, to which living organisms may be critically exposed. Their metabolic and physiological heterogeneity and their complex interspecies interactions are responsible of the survival of bacterial cells in a hostile environment. Moreover, it is well documented that bacteria biofilms show increased resistance to common disinfection methods and are not easily eradicable with conventional antibiotic therapies [2]. Among the most accredited hypotheses of the increased antibiotic resistance is the presence of an extracellular matrix that acts as cell protection and prevents the penetration of the drugs into deeper layers of the biofilm [3]. Therefore, it is mandatory to identify effective strategies for controlling bacteria growth and the related consequent diseases. Bacterial infections in humans are mainly caused by mesophilic bacteria that find their optimal growth temperature around 37 °C, that is the regular human body temperature. However, moderate temperatures (30–45 °C) have been demonstrated to be still suitable to promote mesophilic bacteria growth while higher temperatures (>45 °C) are known to inactivate bacteria viability or proliferation. Therefore, currently, exposure to high temperature is considered a well-established methodology for disinfecting medical tools and laboratory equipment (e.g., by means of dry ovens) [4]. It is obvious that the same highly efficient approach cannot be simply translated for the treatment of human (or animal) diseases caused by pathogenic bacteria, due to the difficulty to locally increase body temperature upon suitable activation.

### Biomedical Applications of Gold Nanoparticles

Nanotechnology offers, due to the design and fabrication of original functional nanomaterials, a powerful weapon against several diseases such as cancer [5,6,7] and pathogenic illness [8,9,10,11]. Particularly, gold nanoparticles (NPs) represent a class of very unique materials, possessing, among other original size dependent properties, the extraordinary intrinsic capability to confine the intense electromagnetic field at the nanoscale. Such a unique feature is enabled due to a physical phenomenon called localized plasmonic resonance (LPR) [12], a coherent and dipolar oscillation of the free electrons localized at the metal/dielectric interface because of the presence of an incident external electromagnetic radiation. Remarkably, the LPR mechanism is associated to a consequent temperature increase, because of the resultant Joule heating effect upon irradiation of a suitable wavelength [13]. Such a feature can pave the way towards the realization of highly efficient nanosized sources of heat [13,14]. Among the different kinds of NPs, colloidal gold nanorods (GNRs), besides the excellent photo-thermal properties, possess unique chemical-physical characteristics related to their specific geometry, that grant them a great potential in a wide range of bio-related applications [15,16,17]. Indeed, colloidal GNRs are not only intrinsically non-toxic and exhibit a promptly processable surface but are also characterized by an optical response extending to the first biological window (700–900 nm), where light has its maximum depth of penetration in biological tissue. GNRs are characterized by two LPR bands, ascribable to the transverse and longitudinal plasmon absorption, typically positioned in the visible and near infrared (NIR) range, respectively, in spectral position dependent on their aspect ratio [12]. Moreover, GNRs are very efficient light to heat converters with an extremely high (≈1) photo-thermal efficiency [14,15,18]. All the aforementioned outstanding properties have represented an incredible breakthrough especially in the fight against cancer through the so-called plasmonic photo-thermal therapy (PPTT) [19,20]. PPTT is a “drug-free” cancer therapy based on the possibility to selectively destroy cancer cells by exploiting the efficient capability of NPs to convert photon energy into heat. In this context, several plasmonic based architectures have been realized with the purpose of photo-thermally inactivate heat-resistant bacteria. Among others, for instance, Norman et al. [21] have used antibodies linked GNRs to selectively destroy gram-negative bacteria. Martinez et al. [22] have reported the minimum inhibitory concentration (MIC) and minimum bactericide concentration (MBC) of GNRs and GNRs combined with PPTT against seven different bacterial strain, observing the ability of GNRs alone to reduce the bacteria population due to the intrinsic toxicity of GNRs (mainly due to the residual free cetyltrimethylammonium bromide (CTAB) employed as a capping agent). In a different approach, Santos et al. [23] have demonstrated a very effective bacterial inactivation technique by depositing bacterial cells onto a random array of nanoporous gold disks. Similarly, Pihl et al. [24] have realized an analogue experiment by using an array of GNRs. In spite of the great potential, such plasmonic platforms cannot be used as a therapy for in-vivo bacteria inactivation. Similarly, NPs solutions to be delivered through the blood stream present several limitations such as intrinsic toxicity [22].

To overcome these issues, here we propose a simple and effective methodology based on capped GNRs, obtained upon a suitable purification from the excess of CTAB used as a capping agent that aims at investigating the potential hold by GNRs in the development of light assisted antimicrobial therapies. To validate the proposed approach, a strain of *Escherichia coli* (*E. coli*) has been selected as a representative type of bacteria, having a great impact as it causes severe illness in humans since it is found in the lower intestine of warm-blooded organisms. In particular, an effective approach has been designed and realized in order to minimize the concentration of GNRs and, accordingly, of CTAB associated to the nanostructures, and still obtaining dispersible GNRs and a sufficiently high temperature (≈50 °C) suited to reduce the *E. coli* population of about 2 log colony-forming unit (CFU). Such a result not only represents an excellent starting point for realizing in vivo light assisted antimicrobial therapies, but also demonstrates to be fully compliant to the typical standard [25] required for antimicrobial treatment in health care facilities. The proposed plasmonic system has been thoroughly investigated highlighting the structure-function relation of their thermo-optical properties and assessing their antimicrobial activity by means of viability assays.

## 2. Materials and Methods

### 2.1. Synthesis and Characterization of Gold Nanorods

Cetyltrimethylammonium bromide (CTAB) capped, water-dispersible, GNRs have been synthesized by means of a seed mediated protocol reported in detail elsewhere [26,27]. Shortly here, we reported that the GNRs samples (C = 3 × 10^−9^ M, determined by utilizing a standard spectroscopic (absorption) technique) were carefully purified from excess of CTAB by several centrifugation cycles in order to reduce the intrinsic cytotoxicity, mainly exhibited by the non-conjugated CTAB. More details on the GNRs purification process and properties are provided in Figure S1 and Table S1 of the Electronic Supplementary Material (ESM) section. Morphological experiments performed with a transmission electron microscopy (TEM, by Jeol JEM-1011 microscope, Jeol, Peabody, MA, USA, operating at 100 kV) indicated that the particle population consisted of GNRs (14 nm × 70 nm) with an aspect ratio of about 5.1. A representative TEM image is reported in the inset of Figure S1 (ESM section). The UV-Vis absorption spectrum (Agilent, Santa Clara, CA, USA, see Figure S1) of GNRs has evidenced the presence of two distinct bands corresponding to the transverse and longitudinal LPR bands centered at 517 nm and 788 nm, respectively.

### 2.2. Effect of Gold Nanorods on Escherichia coli TG1 Growth

*Escherichia coli* TG1 (*E. coli*), kindly provided by Prof. Michele Galluccio (University of Calabria), were selected as gram negative bacterium. *E. coli* were grown aerobically at 37 °C with shaking in sterile Luria Bertani (LB) broth (normal rich growth medium: NaCl 5 g/L; yeast extract 5 g/L; trypton 10 g/L). The *E. coli* solution was diluted (1:100 v/v) in 20 mL of fresh LB liquid medium to restart the cell cycle. After 3 h of incubation at 37 °C the cells were synchronized at the log phase of the growth curve, featured with the optical density at 600 nm (OD600) of 0.4–0.6. At this OD value the growth rate is constant, and the cells are already dividing and result in being metabolically active. GNRs (5.9 μL) were added in 195.5 μL of *E. coli* (C = 10^6^ CFU/mL) so that the final concentration of GNRs was about 8.76 × 10^−11^ M. The culture was kept for 6 h at 37 °C. At 1, 2, 3 and 6 h serial dilutions of the culture were made, and 50 μL of each dilution were cultured onto sterile LB agar (LB broth + agar 16 g/L) and incubated at 37 °C. After 16–18 h, the number of colonies on each plate was determined to get the corresponding concentration of living bacteria. The log N cells were calculated by counting the CFU and using the formula: CFU/mL = (N° of colonies × dilution factor)/volume of the culture spread. The same assay procedures were used as those described above for the strain cultures without GNRs.

### 2.3. Photo-Thermal and Morphological Characterization of Escherichia Coli TG1/Gold Nanorods Solutions

**Figure 1:**
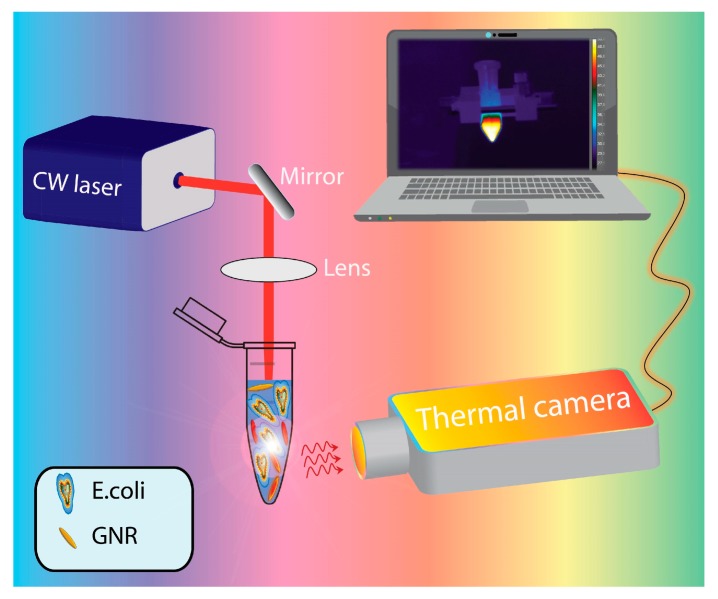
Schematic representation of the thermo-optical setup.

The thermo-optical setup (Figure 1) used for this purpose was based on a CW diode laser (Coherent Inc. Santa Clara, CA, USA; I = 6.3 W/cm^2^) operating at 810 nm in the high absorption region of GNRs spectrum, corresponding to the longitudinal plasmon band (LPR wavelength is centered at 788 nm). The laser beam was focused by means of a 10 cm focal length lens in the central part of the eppendorf tube (spot size ≈ 2 mm). A high-resolution thermal-camera was used for mapping and identifying (side view) both the heating spatial distribution and the temperature profile under top-pumping laser illumination.

The thermographic analysis was carried out by using a FLIR (A655sc) thermal-camera (FLIR System, Wilsonville, OR, USA) that produces thermal images of 640 pixels × 480 pixels with an accuracy of ±2 °C. The camera works seamlessly with proprietary software (FLIR Research IR Max, FLIR System, Wilsonville, OR, USA) that enables recording and processing of the thermal data acquired by the camera. Camera control parameters were set such that sample emissivity was 0.89; camera-sample distance 50 cm and time resolution: 2.67 frame/sec.

Samples for field emission scanning electron microscopy (FE-SEM Zeiss-Sigma, Carl Zeiss Co., Oberkochen, Germany, operating at 10 KV, working distance 3.9 mm) were prepared by dehydrating both *E. coli* and *E. coli*/GNRs solutions on silicon chips, using 70%, 80%, 90%, 95% and 100% ethanol, respectively, for 10 min steps. Subsequently, samples were fixed by using a 3% glutaraldehyde in PBS, pH 7.3 solution and left it to dry overnight. Finally, a thin layer of Au (15 nm) was sputtered just before inserting the samples in the SEM vacuum chamber for reducing the charging effect because of the electrons beam accumulation.

## 3. Experimental Results

**Figure 2:**
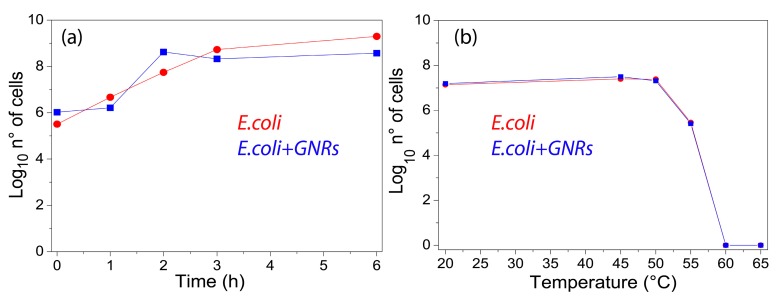
Viability experiments (**a**) without (red curve) and with (blue curve) gold nanorods (GNRs; C = 8.76 × 10^−11^ M) at different incubation times. Viability test (**b**) without (red curve) and with (blue curve) GNRs at different temperatures after 5 min of incubation time.

The toxicity of GNRs was investigated by performing viability experiments, with and without GNRs. Figure 2a shows a comparison of the cells viability between *E. coli* (red curve) and *E. coli*/GNRs (blue curve) at different incubation times. In both cases, regular cells growth was evident, thus pointing out that under the tested experimental conditions cells viability and proliferation did not appear to be significantly affected either by GNRs concentration value and the CTAB molecules present at their surface. A detailed characterization of the *E. coli* viability in the presence of GNRs at different stages of their purification level of residual CTAB is reported in ESM, Figure S2. Thermal treatment experiments were performed in order to investigate the influence of temperature on the cells viability. For this purpose, 500 μL of 1.38 × 10^7^ CFU/mL *E. coli* were deposited on glass slides and temperature was gradually increased from 20 °C to 65 °C by using a hot-plate, without, however, considering the temperature interval from 25 °C to 40 °C, where bacteria are expected to grow.

**Figure 3:**
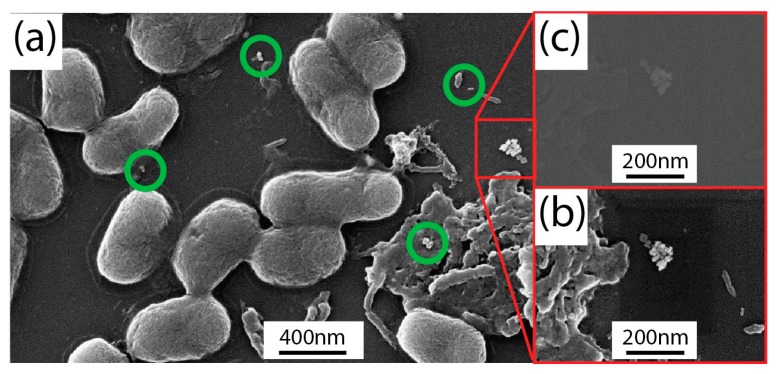
SEM micrograph of the *Escherichia coli*/GNRs solution deposited on a silicon chip (**a**) along with a higher magnification micrograph acquired with the in-lens (**b**) and backscattered electron (BSE) (**c**) detectors, respectively, in split operational mode.

At each scheduled time interval, a defined volume of *E. coli* solution was withdrawn from the glass slide and a series of dilutions (from 10^−1^ to 10^−5^) were made by using the LB broth. Subsequently, 50 μL of each diluted sample was cultured out onto sterile LB agar and incubated at 37 °C to investigate cell viability. Figure 2b (red curve) shows the typical curve of temperature-dependent growth for *E. coli*. As the temperature increased, exceeding the optimum growth temperature (≈37 °C), a decrease in the cell number was observed until their death was noticed at about 60 °C, as caused by the rapid denaturation of proteins. The same experiment performed in the presence of GNRs did not show any significant difference, since the two curves almost overlapped (Figure 2b, blue curve). The influences of GNRs on the *E. coli* morphology were investigated by means of a SEM. In Figure 3a, the SEM micrograph of the *E. coli*/GNRs sample showed the typical elongated shape of *E. coli*. The average longitudinal dimension was 1.4 ± 0.4 µm (for 90 counts), this result was compatible with the values found in a similar investigation performed on dehydrated *E. coli* reported in [28]. In Figure 3a isolated GNRs were evident (green circles and red square) placed quite far away from bacteria. A higher magnification observation performed in correspondence of the red squared region, with both “in-lens” and backscattered electrons (BSEs) detectors, in split mode, resulted in the micrographs reported in Figure 3b,c, respectively. Since the BSE signal strongly depends on the average atomic number (Z) of the specimen, the presence of gold (Z ≈ 80) resulted in a contrast higher than that of the surrounding bacteria (Z ≈ 20) and therefore the GNRs aggregates (Figure 3a, red square) appeared brighter in Figure 3c. Significantly, no GNR was detected at the bacteria surface, while only few GNRs were detected in the area, without any concomitant relevant alteration of *E. coli* morphology induced by the exposure to the GNRs. The presence of only few GNRs (Figure 3a) could be reasonably accounted by the loss of a large fraction of NPs as a consequence of the multiple washing cycles performed with ethanol in order to completely dry the bacteria.

**Figure 4:**
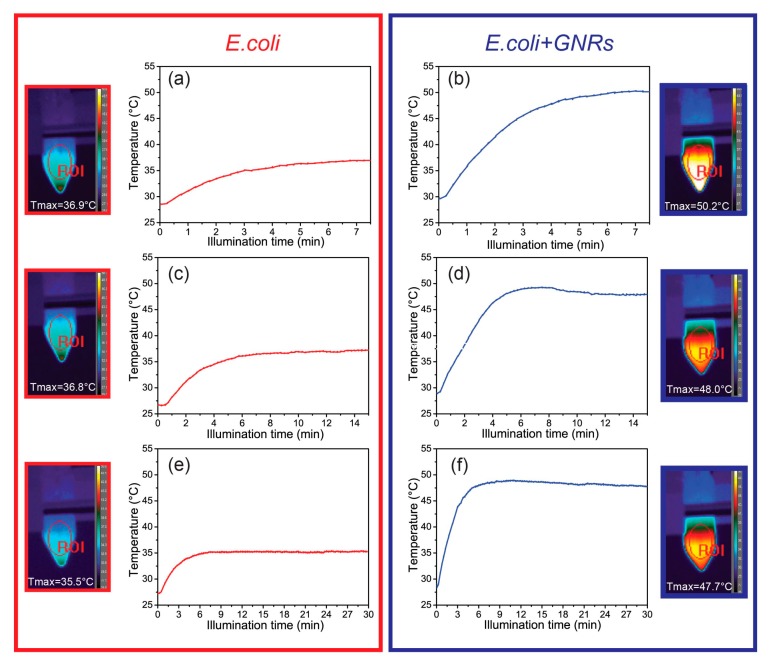
Temperature profiles of the *Escherichia coli* (**a**), (**c**), (**e**) and *E. coli*/GNRs (**b**), (**d**), (**f**) samples for different illumination times along with the corresponding thermographic view of the sample in the eppendorf tube.

Overall the bacterial growth seemed not to be affected by the exposure to GNRs (Figure 2a). Therefore, photothermal experiments were performed to assess whether the GNRs concentration (C = 8.76 × 10^−11^ M) was able to induce a thermo-plasmonic heating suited to affect the *E. coli* population. Figure 4 shows the time-temperature profiles along with the corresponding thermographic pictures at three increasing illumination times (7.5 min, 15 min and 30 min) for both *E. coli* and *E. coli*/GNRs samples. The temperature plots were obtained by selecting, by means of a control software, an elliptical region of interest (ROI), which includes the center of the illuminated area.

Figure 4a,c,e show that upon illumination of the *E. coli* samples, the thermo-camera detected only a relatively low increment of temperature (up to 7–8 °C). Conversely, upon exposure of *E. coli*/GNRs, the absorbed light was efficiently converted into heat because of the plasmonic photo-thermal heating of the GNRs, and the thermo-camera was able to detect a significant temperature increase, as shown in Figure 4b,d,f. Indeed, after 7.5 min, 15 min and 30 min of irradiation, a temperature increase from 20 °C to 50.2 °C, 48.0 °C and 47.7 °C, respectively, were detected that corresponded essentially to the same temperature value, within the experimental variability. The relatively long exposure times (7.5 min, 15 min and 30 min) ensured a uniform distribution of the photo-induced heat (see thermal images reported in Figure 4). Importantly, all the temperature profiles reported in Figure 4 show the same trend. After a rapid temperature increase in the first 5–7 min, a steady state condition was observed. The *E. coli* viability test with and without GNRs, upon laser irradiation, was performed at each scheduled time interval (7.5 min, 15 min and 30 min). Namely, samples obtained upon serial dilutions from each tube were cultured onto LB agar and incubated at 37 °C overnight. Afterwards, the log N cells were calculated by means of the plate count method.

**Figure 5:**
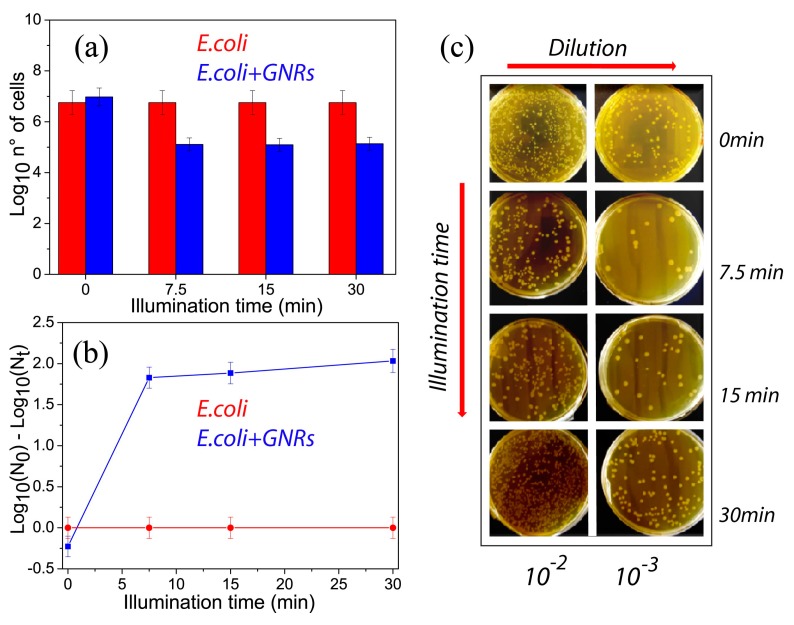
Results of the viability experiments (**a**) carried out with (blue) and without (red) GNRs at different illumination times along with the corresponding killing efficiency (**b**). Pictures of the petri dishes (**c**) containing the *Escherichia coli* /GNRs colonies at increasing illumination times and at different dilution. Data reported in (**a**) were an average of three independent experiments.

The viability results for both *E. coli* (red column) and *E. coli*/GNRs (blue column) samples (Figure 5a) clearly pointed out that the laser irradiation did not affect the viability of *E. coli* even after 30 min of continuous illumination (Figure 5b (red curve)). Conversely, a significant increase of the killing efficiency (defined as the difference between the bacteria population at t = 0 min and t = 7.5 min), of about 2 log CFU, was observed in the presence of GNRs as shown in Figure 5b (blue curve). It is worth pointing out that the data reported in Figure 5a were normalized for every experiment to the bacterial population at each specific time interval in order to take into account the intrinsic cells proliferation.

Moreover, it is important to point out that the protocol followed in the experiments for bacteria growth and counting were highly standardized. Indeed, the bacteria population at different illumination times was confirmed to be essentially the same (Figure 5a, red histogram plot), thus highlighting the capability to control the bacteria growth with high reproducibility. This, finally, turns in the same deviation, and hence in the same extent of the error bars for the results reported in Figure 5a and for those reported in Figure 5b.

In order to be able to reliably count the *E. coli* colonies, the samples were diluted with LB broth and the pictures of the plates obtained from the exposed solutions as prepared and after dilution, in the presence of GNRs, is reported in Figure 5c. The plate count method was successfully applied on the cultured diluted samples (Figure 5c, right row), where well-distinguished colonies were present, while the high-density colonies visible in the cultures of the less diluted samples (Figure 5c, left row) resulted in being difficult to be safely counted.

For the sake of simplicity, photos of the petri dishes containing the *E. coli* culture without the GNRs at different illumination times and dilution factors were corresponding to the culture with GNRs at t = 0 min. It is important to point out that in order to clarify whether the light driven thermo-plasmonic process explored so far was bacteriostatic or bactericidal, a more careful analysis of the experimental data reported in Figure 5a is required. It turns out that in the presence of GNRs there was a reduction of about 2 log CFU of *E. coli* population (higher than 90% in terms of viability reduction) after 7.5 min of laser illumination. On the other hand, no further reduction in the *E. coli* population was observed after 15 min and 30 min of laser exposure, in the presence of GNRs. This behavior could be explained by considering that after 15 min the bacterial cells had already implemented the molecular response mechanisms to environmental stress such as the plasmonic assisted photo-thermal heating with possible DNA lesions and thus adapted to the new external conditions [28,29]. As a consequence, we can safely state that up to 7.5 min of laser irradiation the light assisted thermo-plasmonic heating resulted in a bactericidal effect, in agreement with the accepted definition of bactericidal property [30]. After 7.5 min of laser illumination, the bacteria population remains constant (Figure 5b, blue curve). This evidence suggests that the conversion of light to heat due to the presence of highly efficient nano-converters (GNRs) ensures a bacteriostatic condition. Furthermore, in order to confirm a long term-effect of the bacteriostatic condition, we performed a comparison between the bacteria population immediately and several hours after the end of the illumination process, being in the meanwhile the solution was incubated at 37 °C (data not shown because were very similar to that reported in Figure 5a). No change in the bacteria population had been measured.

## 4. Discussion

Currently plasmonic NPs are investigated in several research fields such as electronics [31], photonics [32], agriculture [33], medicine [34] and environmental remediation [35]. As far as their application in medicine, they have successfully demonstrated their potential in cancer therapy and treatment of pathogenic diseases [21,22]. In particular, the use of plasmonic NPs for treatment of pathogenic diseases is a relatively new and promising field, since most of the efforts reported so far indicate several advantages, including the possibility of targeting and delivering. However, a number of unknown side effects, including toxicity, were described. Also, in vivo experiments require the use of colloidal solutions of NPs, suited to be delivered to the targeted sites via blood stream or in situ injections. Here, the study evaluated the possibility to treat *E. coli* by taking advantage of the photo-thermal properties of organic capped GNRs, since they are extremely stable, possess an intrinsic low toxicity and can be promptly functionalized. Indeed, the optical response of the GNRs in the first biological spectral window along with their outstanding photons to thermal-energy conversion property, make them ideal candidates for this purpose [14,15,18]. Similar approaches have been reported based on metal nanostructures [21,22,23]. However, the intrinsic toxicity of NPs or, alternatively, the use of rigid functional platforms result was not amenable for applications. Therefore, here water dispersible CTAB capped GNRs, synthesized by means of a simple colloidal approach were used. CTAB, the surfactant used in GNRs synthesis, plays an important role controlling the GNRs size distribution, providing them colloidal stability and preventing NP aggregation. On the other hand, CTAB has been reported to result, above a given concentration, in being very harmful in terms of cells viability [36]. Therefore, the “as prepared” GNRs were purified in order to remove excess of CTAB, that is the fraction of the surfactant molecules that only loosely bind the surface of the GNRs that, however, still result in being sufficiently capped to retain their colloidal stability. Subsequently the work has investigated the suitable conditions, namely the proper concentration of GNRs able to ensure bacterial viability and, at the same time, enable the access to their photo-thermal properties. The results of the comprehensive viability set of experiments performed to this end, have demonstrated that while the CTAB capped GNRs, at the investigated concentration, did no significantly affect the cells viability and proliferation (Figure 2a). GNRs were able to significantly reduce the bacterial viability only upon light irradiation. Such a result represents a significant advance with respect to the current findings [22], that accounts for reporting the ability of GNRs to reduce the bacterial population, relying, however, only on the inherent toxicity of GNRs, without exploiting their ability to photo-thermally induce heating. Here, the photo-thermal experiments have shown that GNRs (not cytotoxic concentration, C = 8.76 × 10^−11^ M) were able, upon a suitable laser illumination, to increase a temperature of about 20 °C (Figure 4b,d,f). Conversely, under the same laser irradiation (Figure 4a,c,e) in the absence of GNRs, only a relatively low increment of temperature (up to 7–8 °C) had been detected. This variation turned out to be completely harmless in terms of bacterial viability. It is important to mention that bacteria viability experiments were performed at two distinct GNRs concentration (C = 2.92 × 10^−11^ M and C = 8.76 × 10^−11^ M), however, although any toxicity effect had been observed for the two concentration values, different photo-thermal properties were detected, resulting in a weaker effect for 2.92 × 10^−11^ M. Therefore the 8.76 × 10^−11^ M GNRs concentration were then used in the investigation. The obtained results, highlighting the extraordinary capability of GNRs to efficiently convert a NIR radiation into heat, showed that the dynamic of the system was very fast, as indicated by the time-temperature profiles reported in Figure 4a,c,e. These characteristics appear particularly advantageous for the therapeutic potential of the system, as the temperature of about 50 °C was found to be reached in less than 5 min (Figure 4b,d,f)). A set of viability experiments systematically performed on the *E. coli* culture upon laser irradiation (Figure 5b) have demonstrated a photo-thermal killing efficiency suited to reduce 2 log CFU the cell number after 7.5 min. Significantly, the outcome of the viability tests performed on the system that was photo-thermally heated at 50 °C (Figure 5b) resulted in a much higher reduction of the bacteria viability that found for the system heated up to the same temperature (Figure 2b, blue curve). Such a discrepancy could be explained by considering that the photo-thermal heating due to the presence of the highly efficient nano-sources of heat represented by the GNRs, induces a much higher localized temperature variations, while the thermal-camera is able to only detect the temperature variations at the glass/air interface of the eppendorf tube. Moreover, the heat generation at the nanoscale was followed by other secondary mechanisms such as the formation of sound waves and generation of nano-bubbles, which, in their turn, may contribute to the *E. coli* killing. The remarkable achievement of a killing efficiency of 2 log CFU in a short time interval (7.5 min.) could be improved even more, by engineering the GNRs properties in terms of surface chemistry functionalization, aspect ratio and concentration, thus possibly further increasing the temperature to value (e.g., 60 °C) sufficiently high enough to completely kill all the living bacteria (Figure 2b), therefore representing a real breakthrough towards the realization of light assisted antimicrobial technologies.

## 5. Conclusions

An all-optical methodology based on the capability of GNRs to convert a NIR radiation into heat for an antibacterial application was reported. As a proof-of-concept, *E. coli*, selected as a representative microorganism, was treated with water dispersible, conveniently purified CTAB capped, GNRs, by exposing the *E. coli* culture to GNRs aqueous solution at a suitable, not cytotoxic concentration level. Cell viability assays demonstrated that the GNRs, under the investigated concentration, did not affect cells viability and proliferation. The SEM analysis on the investigated *E. coli*/GNRs culture, after a dehydration process, clearly accounted for the bacteria morphology, while the backscattered electrons (BSE) micrograph confirmed that the GNRs result was not in contact with bacteria. Photo-thermal experiments carried-out with a laser light (λ = 810 nm) assisted thermographic setup pointed out that by irradiating the GNRs in the *E. coli*, the temperature could be increased up to 50 °C in about 5 min, and cells viability experiments demonstrated that, under such a laser light irradiation (I = 6.3 W/cm^2^) the presence of GNRs in the *E. coli* culture was able to induce a suppression of living bacteria of about 2 log CFU. A further optimization of the nanoparticles properties in terms of biocompatibility and molecular recognition capability (e.g., using proteins, antibodies, peptides, etc.) is a mandatory step for realizing a new generation of light assisted and on-demand antimicrobial therapies.

## Supplementary Materials

The following are available online at https://www.mdpi.com/1996-1944/12/9/1530/s1, Figure S1: Normalized spectral response of the samples along with a representative TEM image, Table S1: Zeta-potential of the samples along with the wavelengths of the transverse and longitudinal LPR peaks, Figure S2: Viability experiments without (green curve) and with (red, blue and magenta curves) the three different GNRs solutions at different incubation times.

## Abbreviations

The following abbreviations are used in this manuscript:

PPTT	Plasmonic photo-thermal therapy
NPs	Gold nanoparticles
PPTT	Plasmonic photo-thermal therapy
GNRs	Gold nanorods
LPR	Localized plasmonic resonance
MIC	Minimum inhibitory concentration
MBC	Minimum bactericide concentration
NIR	Near infrared
E. coli	Escherichia coli TG1
