# Supplementary material for: Thermo-Plasmonic Killing of Escherichia coli TG1 Bacteria
Source: Materials (Basel). 2019 May 10;12(9):1530. doi: 10.3390/ma12091530 (PMC6539421; doi:10.3390/ma12091530)
Supplement: Supplementary file 1 [file materials-12-01530-s001.pdf]

materials

Thermo-Plasmonic Killing of *Escherichia coli* TG1 Bacteria

Ferdinanda Annesi [1,†], Alfredo Pane [1,†], Maria Adele Losso [2], Alexa Guglielmelli [3,1], Fabrizio Lucente [2], Francesca Petronella [4], Tiziana Placido [4], Roberto Comparelli [4], Maria Grazia Guzzo [2], Maria Lucia Curri [4,5], Roberto Bartolino [3,1] and Luciano De Sio [6,1,*]

[1] CNR-Lab. Licryl, Institute NANOTEC, Arcavacata di Rende, 87036, Italy; ferdinanda.annesi@cnr.it (F.A.); alfredo.pane@cnr.it (A.P.); alexa.guglielmelli@unical.it (A.G.); roberto.bartolino@fis.unical.it (R.B.)
[2] Department DiBEST (Biology, Ecology and Earth Sciences), University of Calabria, Arcavacata di Rende, 87036, Italy; maria_adele.losso@unical.it (M.A.L.); fabrizio.lucente93@gmail.com (F.L.); mariagraziaguzzo22@gmail.com (M.G.G.)
[3] Department of Physics, University of Calabria, 87036, Arcavacata di Rende, Cosenza 87036, Italy
[4] CNR-IPCF, National Research Council of Italy, Institute for Physical and Chemical Processes-Bari Division, Via Orabona 4, Bari, I-70126, Italy; f.petronella@ba.ipcf.cnr.it (F.P.); t.placido@ba.ipcf.cnr.it (T.P.); r.comparelli@ba.ipcf.cnr.it (R.C.); lucia.curri@ba.ipcf.cnr.it (M.L.C.)
[5] Department of Chemistry, "A. Moro" University of Bari, Via Orabona 4, I-70126 Bari, Italy
[6] Department of Medico-surgical Sciences and Biotechnologies, Sapienza University of Rome, Corso della Repubblica 79, 04100, Latina, Italy
† These authors contributed equally to this work
* Correspondence: luciano.desio@uniroma1.it; Tel.: +39-077-317-57217

Electronic Supplementary Materials

Gold nanorods (GNRs) were purified from excess of CTAB by several centrifugation cycles in order to reduce the intrinsic cytotoxicity, mainly exhibited by the non-conjugated CTAB. To do so, we have prepared three different GNRs solutions labeled A, B, C trough subsequent post-synthesis washing/centrifugation cycles at 6000 rpm, using MilliQ water as a washing agent. In particular, the solutions A, B and C were obtained from the as synthesized [1,2] GNRs upon 1, 2 and 3 washing/centrifugation cycles, respectively, with the goal to progressively remove the non-conjugated CTAB molecules used to stabilize the GNRs surface. In this respect, the three solutions were step by step characterized by absorption spectroscopy and their surface charge was measured by Z-potential analysis.

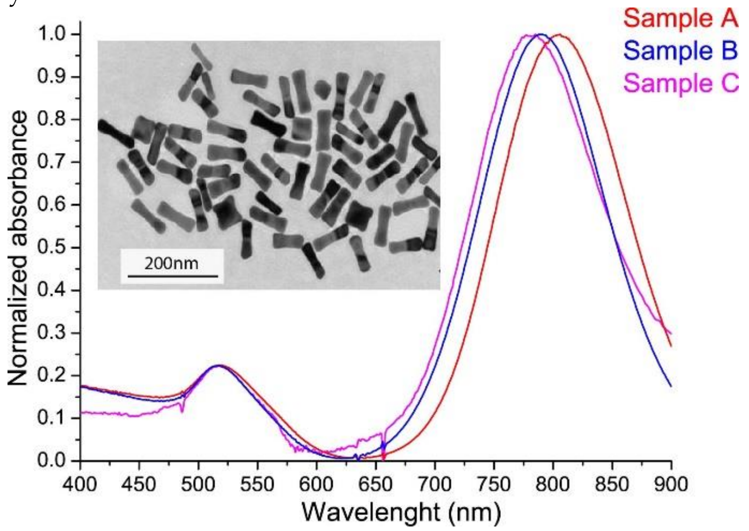

Figure 1. Normalized spectral response of the samples along with a representative TEM image (inset).

Table 1. Zeta-potential of the samples along with the wavelengths of the transverse and longitudinal localized plasmonic resonance (LPR) peaks.

Sample	Zeta potential (mV)	Transverse LPR peak (nm)	Longitudinal LPR peak (nm)

A	27.7	517	804
B	9.7	517	788
C	-6.3	517	779

The absorption spectra of the three solutions (C = $3x10^{-9}$ M) reported in Figure S1 evidenced the presence of two distinct bands corresponding to the transverse and longitudinal LPR peaks. Particularly, the longitudinal peak exhibited a progressive blue shift from 804 nm (Figure S1, sample A, red curve) to 779 nm (Figure S1, sample C, magenta curve) while the transverse peak did not exhibit any relevant shift. This behavior could be explained by considering that the optical properties of ellipsoidal particles were predicted using the Gans theory [3] framework, which describes the optical properties of ellipsoidal NPs as based on the dipole approximation. The wavelength shift could be explained by taking into account that the longitudinal band of GNRs is very sensitive to the refractive index of the medium surrounding the GNRs. In the actual case, by progressively removing the residual CTAB there is a gradual reduction of the average refractive index surrounding the GNRs. The blue shift of about 25 nm (see Table S1) was consistent with the removal of residual CTAB. Indeed, the refractive index of CTAB was 1.4 while that of water was 1.33. This evidence is in agreement with previously reported results [4]. Due to the low sensitivity to refractive index variations, the transverse band did not exhibit any visible shift; thus, this behavior highlights the high sensitivity of the longitudinal LPR band to variation of the refractive index of the surrounding medium. Figure S1 (inset) shows a representative TEM image: It indicates that the particle population consisted mainly of GNRs with a 5.1 ± 0.3 aspect ratio. The Z-potential values reported in Table S1 evidenced a variation of the electrokinetic charge from +27.7 mV (sample A) to –6.3mV (sample C), confirming the removal of the positive charged CTAB molecules.

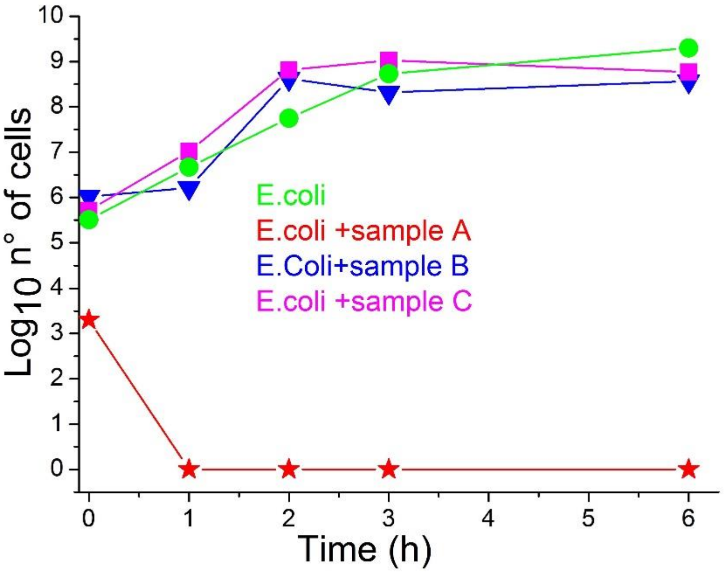

Figure 2. Viability experiments without (green curve) and with (red, blue and magenta curves) the three different GNRs solutions at different incubation times.

To verify the cytotoxicity effect of the three GNRs solutions, cell viability experiments were performed with and without GNRs (C = 8.76 × 10^{-11} M). Figure S2 shows a comparison of the cells viability between the *E. coli* (green curve) and *E.coli*/GNRs (red, blue and magenta curves) at different incubation times. Results evidenced that, although the first purification cycle, sample A (Figure S2, red curve) still exhibited a very high cytotoxicity due to the high concentration of residual CTAB. Samples B and C did not affect (at all) both cells viability and proliferation. As evidenced, the bacterial growth curves (Figure S2, blue and magenta curves) exhibited the same behavior of the bacterial growth curve obtained without GNRs (Figure S2, green curve). It is important to say that for our experiments we have used the sample labeled B because it exhibited both low concentration of residual CTAB and positive Z-potential, ensuring both negligible toxicity and high colloidal stability.

References

1. Placido T, Aragay G, Pons J, Comparelli R, Curri ML, Merkoçi A. Ion-Directed Assembly of Gold Nanorods: A Strategy for Mercury Detection. *ACS Applied Materials & Interfaces*, 5(3), 1084-1092 (2013).
2. De Sio L, Placido T, Serak S *et al.* Nano-Localized Heating Source for Photonics and Plasmonics. *Advanced Optical Materials*, 1(12), 899-904 (2013).
3. Gans R. Über die Form ultramikroskopischer Goldteilchen. *Annalen der Physik*, 342(5), 881-900 (1912).
4. He J, Unser S, Bruzas I *et al.* The facile removal of CTAB from the surface of gold nanorods. *Colloids and Surfaces B: Biointerfaces*, 163, 140-145 (2018).
